# Bile Acids and Dysbiosis in Non-Alcoholic Fatty Liver Disease

**DOI:** 10.1371/journal.pone.0151829

**Published:** 2016-05-20

**Authors:** Marialena Mouzaki, Alice Y. Wang, Robert Bandsma, Elena M. Comelli, Bianca M. Arendt, Ling Zhang, Scott Fung, Sandra E. Fischer, Ian G. McGilvray, Johane P. Allard

**Affiliations:** 1 Department of Pediatrics, Division of Gastroenterology, Hepatology and Nutrition, The Hospital for Sick Children, Toronto, ON, Canada; 2 Physiology and Experimental Medicine Program, Research Institute, The Hospital for Sick Children, Toronto, ON, Canada; 3 Department of Nutritional Sciences, University of Toronto, Toronto, ON, Canada; 4 Centre for Child Nutrition Health and Development, University of Toronto, Toronto, ON, Canada; 5 Toronto General Hospital, University Health Network, Toronto, ON, Canada; 6 Department of Medicine, University of Toronto, Toronto, ON, Canada; 7 Department of Laboratory Medicine and Pathobiology, University of Toronto, Toronto, ON, Canada; 8 Department of Surgery, University of Toronto, Toronto, ON, Canada; University of Basque Country, SPAIN

## Abstract

**Background & Aims:**

Non-alcoholic fatty liver disease (NAFLD) is characterized by dysbiosis. The bidirectional effects between intestinal microbiota (IM) and bile acids (BA) suggest that dysbiosis may be accompanied by an altered bile acid (BA) homeostasis, which in turn can contribute to the metabolic dysregulation seen in NAFLD. This study sought to examine BA homeostasis in patients with NAFLD and to relate that with IM data.

**Methods:**

This was a prospective, cross-sectional study of adults with biopsy-confirmed NAFLD (non-alcoholic fatty liver: NAFL or non-alcoholic steatohepatitis: NASH) and healthy controls (HC). Clinical and laboratory data, stool samples and 7-day food records were collected. Fecal BA profiles, serum markers of BA synthesis 7-alpha-hydroxy-4-cholesten-3-one (C4) and intestinal BA signalling, as well as IM composition were assessed.

**Results:**

53 subjects were included: 25 HC, 12 NAFL and 16 NASH. Levels of total fecal BA, cholic acid (CA), chenodeoxycholic acid (CDCA) and BA synthesis were higher in patients with NASH compared to HC (*p*<0.05 for all comparisons). The primary to secondary BA ratio was higher in NASH compared to HC (*p =* 0.004), but ratio of conjugated to unconjugated BAs was not different between the groups. Bacteroidetes and *Clostridium leptum* counts were decreased in in a subset of 16 patients with NASH compared to 25 HC, after adjusting for body mass index and weight-adjusted calorie intake (*p* = 0.028 and *p* = 0.030, respectively). *C*. *leptum* was positively correlated with fecal unconjugated lithocholic acid (LCA) (*r* = 0.526, *p* = 0.003) and inversely with unconjugated CA (*r* = -0.669, *p*<0.0001) and unconjugated CDCA (*r* = - 0.630, *p*<0.0001). FGF19 levels were not different between the groups (*p* = 0.114).

**Conclusions:**

In adults with NAFLD, dysbiosis is associated with altered BA homeostasis, which renders them at increased risk of hepatic injury.

## Introduction/Background

Non-alcoholic fatty liver disease (NAFLD) is a term used to describe a spectrum of liver pathologies ranging from hepatic steatosis, non-alcoholic steatohepatitis (NASH) to cirrhosis [1]. As the hepatic manifestation of metabolic syndrome, NAFLD is also closely associated with obesity, diabetes, dyslipidemia and coronary artery disease [2]. NAFLD is currently the most prevalent form of chronic liver disease in both adults and children and its prevalence is rapidly increasing [3]. In adults, it is also the second most common indication for liver transplantation, exerting a major health and financial burden [4].

Despite its prevalence, the development and progression of NALFD is not entirely understood. A growing body of evidence has suggested that changes in intestinal microbiota (IM) may promote NAFLD, through their effects on nutrient digestion and absorption, appetite regulation, host gene expression and immune function [5, 6]. The human intestinal lumen hosts trillions of microorganisms, including bacteria, Archaea, viruses and fungi [7]. While inter-individual IM variation exists, the human IM is dominated by two major phyla: Bacteroidetes and Firmicutes [8]. We and others have demonstrated that NAFLD is characterized by dysbiosis independent of obesity, as evidenced by altered counts of Bacteroidetes and Proteobacteria, as well as small intestinal bacterial overgrowth [9–12].

The impact of IM on the development and progression of NAFLD may be mediated in part through bile acids (BA). BA are a subject of growing interest for their ability to act as signaling molecules, in addition to their role in lipid solubilization and digestion [13]. They regulate hepatic glucose and lipid metabolism, inflammation, as well as their own synthesis through the activation of various nuclear receptors (NRs), such as the farnesoid X receptor (FXR) [14, 15]. Synthesized from cholesterol and conjugated in the liver, BA are modified in the gut by IM and subsequently reabsorbed, in a process called enterohepatic circulation. The IM can change the size and composition of the BA pool through their effects on BA metabolism, namely synthesis, deconjugation and conversion of primary to secondary BA [16, 17]. Reciprocally, BA may also exert antimicrobial activities through their detergent effects on bacterial cell membranes, altering the composition of the IM [18].

BA metabolism in the setting of NAFLD has not been studied comprehensively and it is currently not known whether patients with NAFLD have a different fecal BA composition than healthy subjects [19–21]. The interplay between BA and IM in human NAFLD has not been investigated either. Exploring the interactions between IM and BA in NAFLD can not only further our understanding of the pathogenesis of this condition, but it can also provide direct targets for treatment.

The aims of this study were to 1. Assess and compare the fecal BA profiles of adults with biopsy-confirmed non-alcoholic fatty liver (NAFL), NASH and healthy controls (HC); 2. Compare the rates of hepatic BA synthesis between patients with NAFL, NASH, as well as HC; and 3. Explore associations between fecal BA profiles and IM composition. We hypothesized that patients with NASH have a unique BA homeostasis that places them at higher risk of liver injury compared to those with NAFL or HC.

## Materials and Methods

This was a prospective, cross-sectional study conducted at the University Health Network, Toronto, Canada. The study protocol adhered to the ethical guidelines of the 1975 Declaration of Helsinki and was approved by the institutional review board of the University Health Network and the Hospital for Sick Children in Toronto, Ontario, Canada. All participants provided informed consent in writing.

### Subjects

Patients suspected to have NAFL and NASH were recruited from hepatology clinics and were assessed as per standard of care to rule out other causes of liver disease. Patients with NAFLD were eligible for recruitment if they had had a liver biopsy confirming NAFL or NASH. Liver biopsies were typically performed following 3–6 months of persistently elevated alanine aminotransferase (ALT) levels.

HC subjects were recruited from the Living Donor Transplant Program at the University Health Network. Health control subjects were rigorously assessed according to the Transplant Program’s protocol to ensure they had no significant medical comorbidities and the healthy status of their livers was confirmed with liver biopsies obtained at the time of donation.

Exclusion criteria for both patients with NAFLD and HC were the presence of other liver diseases, end-stage liver disease, anticipated liver transplant within 12 months, chronic gastrointestinal illnesses, history of gastrointestinal operation modifying the intestinal anatomy, use of medications associated with steatosis/steatohepatitis (e.g. corticosteroids), vitamin E or fish oil supplements; consumption of more than 20g of alcohol per day; pregnancy or lactation.

After providing informed consent, all subjects received instructions on how to collect and transport their stool samples and how to complete 7-day food records. The food records were reviewed with the patients following completion, to enhance the quality of the data.

### Clinical data and nutrition assessment

Subjects completed the 7-day food record during the week prior to liver biopsy (or liver donation). Portion sizes were estimated using the 2D Food Portion Visual chart (Nutrition Consulting Enterprises, Framingham, MA) and food records were analyzed for macro- and micronutrient content using Food Processor SQL, Version 7 (ESHA Research, Salem, OR). Patients were asked not to change their diets for the purposes of the study.

### Biochemistry

Fasting glucose was measured by the enzymatic hexokinase method on an Architect c8000 System (Abbot Laboratories, Abbot Park, IL). Serum insulin was determined by radioimmunoassay (Immulite 2500, Siemens Diagnostics, Los Angeles, CA). Hemoglobin A1c in plasma was measured by ion exchange HPLC (Variant II analyzer, Bio-Rad Laboratories, Montreal, QC, Canada). ALT, aspartate aminotransferase (AST), and alkaline phosphatase (ALP) in plasma, as well as fasting serum triglycerides and total cholesterol were measured using the Architect c8000 system (Abbot Laboratories). Serum biochemistry was performed by the diagnostic testing laboratory at University Health Network, Toronto.

### Histology

A pathologist blinded to clinical characteristics assessed liver histology for presence of steatosis, inflammation, and fibrosis. The presence of NASH was determined using the Brunt scoring system, which is a validated and reproducible tool for the evaluation of NASH [22]. The NAFLD activity score (NAS) was also determined [23]

### Quantification of fecal BA

Bile acid quantification was performed by the Sick Kids Analytical Facility for Molecules (AFBM) by liquid chromatography tandem mass spectrometry (LC-MS-MS) using the Biocrates® Life Sciences Bile Acids Kit (Biocrates, Innsbruck, Austria) according to the manufacturer’s instructions. The analysis was performed using an Agilent UHPLC 1290 LC system coupled to an ABSciex QTRAP 5500 in negative ESI MRM mode. The samples were lyophilized, reconstituted with 75% ethanol and homogenized. After mixing by vortex for 5 minutes, the homogenates were centrifuged at 20,000 g for 10 minutes. The supernatants were transferred into new tubes for bile acid determination.

### Quantification of 7-alpha-hydroxy-4-cholesten-3-one (C4)

Plasma 7-alpha-hydroxy-4-cholesten-3-one (C4) concentrations were measured as a marker of bile acid synthesis. C4 measurement was done by Liquid chromatography–tandem mass spectrometry (LC-MS-MS). C4 was extracted by the salting-out method [24, 25]. One hundred μL of serum were diluted with 200 μL distilled water in a 1.5 mL Eppendorf microcentrifuge tube. Five ng of d7-C4 (used as internal standard) and 500 μL of acetonitrile were then added. After the addition of ~100 mg of ammonium sulfate, tubes were vortexed for 1 minute and centrifuged at 2,000 g at 4°C for 5minutes. The supernatant acetonitrile phase was collected and dried down under nitrogen at 35°C. The residues were reconstituted with 200 μL methanol and then vortexed for 1 minute. Next, the samples were transferred into a set of 1.5 mL Eppendorf microcentrifuge tubes and left to incubate for 10 minutes. After centrifugation at 20,000 g for 5 minutes, clear supernatants were transferred to a set of 250 μL inserts for LC-MS-MS analysis. The LC-MS-MS system consisted of an Agilent UHPLC 1290 LC system coupled to an ABSciex QTRAP 5500 in positive ESI MRM mode. Chromatographic separation was performed using a XB-C18 kinetex column (50 x 3.0 mm, 2.6 μm; Phenomenex) operated at a flow rate of 800 μL/minute and eluted isocratically with a mobile phase consisted of acetonitrile/water (98/2, v/v) containing 0.1% trifluoroacetic acid. The total analysis time was 2.5 minutes. Quantitation of C4 was carried out by comparing the deuterium-to-protium ratio of the samples with a standard line generated from authentic C4 using the Analyst 1.6 software. Authentic standards in appropriate dilutions (0–100 ng) mixed with 5 ng of d7-C4 were prepared, and the standard curve of C4 was extracted and analyzed at the same time as the samples.

### Quantification of fibroblast growth factor 19 (FGF-19)

Serum FGF-19 levels were studied as a marker of FXR activation. FGF-19 concentrations were measured using sandwich ELISA kit (Human FGF19 Quantikine ELISA kit, R&D Systems). Samples were diluted (1:1.2) using the Calibrator Diluent RD5P (1X) supplied with the kit. The assay was performed using 100 μl of each sample according to the manufacturer’s instructions.

### Stool sample collection and quantification of intestinal microbiota

Patients collected one stool sample within 24 hours of their clinic appointment using a stool collection kit, which consisted of a plastic collection/storage container with a tightly closing lid, an insulated bag, and cooling elements. Patients immediately froze their samples in their home freezer (-20°C) and transported the sample to the hospital using the cooling elements and the insulated bag, similar to previously published methods [26]. Stool samples were stored at -80°C until analysis. The stool was thawed and immediately homogenized with a masticator blender. 0.1 g was used for DNA extraction using the E.Z.N.A. stool DNA Isolation Kit (Omega, Norcross, GA), according to the manufacturer’s protocol. A lysozyme digestion step (incubation at 37°C for 30 minutes) was added to the extraction protocol. DNA concentration and purity were measured using ThermoScientific Nanodrop 1000 Spectrophotometer (ThermoScientific, Rockford, IL). DNA samples were subsequently stored at -20°C. Fifty nanograms of the extracted DNA were used for the quantification of fecal bifidobacteria, Bacteroides/Prevotella, *Clostridium leptum*, *C*. *coccoides*, *Escherichia coli*, as well as total bacteria and Archaea, by quantitative polymerase chain reaction (qPCR) using a 7900HT thermocycler from Applied Biosystems (Foster City, CA) under default thermocycling conditions. Custom-made TaqMan primers for total bacteria [27], *C*. *coccoides* [27], *C*. *leptum* [27], Bacteroides/Prevotella [27, 28], bifidobacteria [27], and Archaea [29] were used. SYBR Green Gene Expression master mix (Applied Biosystems) and the specific forward and reverse primer were used to conduct real-time PCR for *E*. *coli* [27]. Number of cells of each microorganism in fecal samples was calculated by interpolation from standard curves and expressed as log cell counts/g feces. Bacteroides/Prevotella counts were considered representative of the Bacteroidetes phylum (as previously) and will herein be referred to as Bacteroidetes [28].

### Statistical analyses

Data is presented as median (range). All statistical analyses were conducted with SPSS v.22 (IBM Corp. 2013) and GraphPad Prism v. 4.0 (GraphPad Software, La Jolla, CA). Comparison between HC, NAFL and NASH groups were conducted using the one-way ANOVA, with Tukey’s post hoc test for multiple comparisons or Kruskal-Wallis test according to the distribution of data. Correspondingly, Spearman or Pearson’s correlation coefficient was used for correlations. Statistical significance was accepted at *p* < 0.05.

## Results

### Demographic and laboratory data

A total of 53 subjects were included in this study: 25 HC, 12 patients with NAFL and 16 with NASH. Their demographic, laboratory and nutrition data are summarized in **Table 1**. Patients with NAFL and NASH were older than HC. As expected, patients with NASH had a higher BMI compared to HC; interestingly, the median BMI of HC was in the overweight range. Patients with NASH had a lower weight-adjusted reported energy intake (kcal/kg/day) compared to HC and were more likely to have IR, as shown by higher Homeostasis Model Assessment of Insulin Resistance (HOMA-IR), Hemoglobin A1c (HbA1c) and triglyceride levels in this group (**Table 1**).

**Table 1:** Demographic and laboratory results.

Variables	HC (n = 25)	NAFL (n = 12)	NASH (n = 16)
Age (years)	37 ± 10^a,b^	45 ± 9^a^	48 ± 12^b^
Gender (% male)	56	67	50
BMI (kg/m^2^)*	27 (20–35)^a^	28 (25–44)	33 (24–50)^a^
Waist circumference (cm)**	90 (73–108)^a^	97 (87–122)	107 (89–120)^a^
Hip circumference (cm)	103 (87–120)	106 (96–123)	107 (100–123)
Hypertension (%)	0	8	6
ALT (U/L)**	18 (7–38)^a,b^	42.5 (14–116)^a,c^	84 (39–168)^b,c^
AST (U/L)**	19 (12–29)^a^	26 (16–53)	46 (23–114)^a^
ALP (U/L)	64 (42–130)	68 (40–101)	71 (37–107)
Glucose (mmol/L)	4.9 (4.0–6.5)	5.3 (4.6–11.4)	5.7 (4.1–7.5)
Insulin (pmol/L)*	40 (15–244)^a^	33 (15–465)	102 (38–720)^a^
HOMA-IR*	1.3 (0.5–7.6)^a^	1.6 (0.6–15.6)	4.3 (1.2–40.0)^a^
Hemoglobin A1c %*	5.1 (4.4–7.2)^a,b^	5.3 (0.1–5.8)^a^	5.9 (5.3–7.3)^b^
Triglycerides (mmol/L)*	0.9 (0.5–3.3)^a^	0.9 (0.6–1.8)^b^	1.8 (0.8–5.9)^a,b^
Total cholesterol (mmol/L)	5.05 (3.57–6.69)	5.64 (3.88–7.03)	4.64 (2.65–9.83)
High density lipoprotein (HDL) (mmol/L)	1.2 (0.8–2.0)	1.3 (1.0–1.7)	1.1 (0.3–1.5)
Low density lipoprotein (LDL) (mmol/L)*	3.2 (1.9–4.7)	3.8 (2.2–5.1)^a^	2.8 (1.3–4.2)^a^
Energy intake (kcal/day)	1784 (1246–2862)	1814 (997–2504)	1684 (737–2717)
Fat-derived energy %	36 (23–47)	29 (19–41)	32 (7–49)
Carbohydrate-derived energy %	47 (38–62)	48 (41–58)	47 (25–85)
Weight-adjusted energy intake (kcal/kg/day)*	24.4 (16.0–48.8)^a^	22.7 (11.9–36.8)	18.3 (7.6–35.0)^a^

The values are expressed as medians (range).

* *p* < 0.05.

** *p* < 0.001.

For each comparison, identical letters indicate the groups between which the statistical difference was significant.

### Fecal bile acids

**Fig 1:**
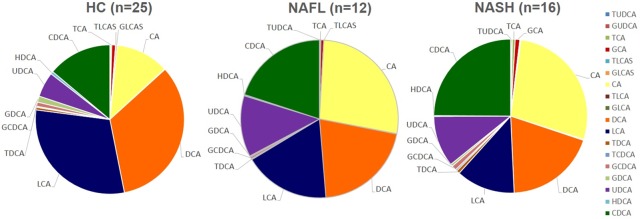
Changes in fecal bile acid composition in HC, NAFL and NASH patients.

**Fig 2:**
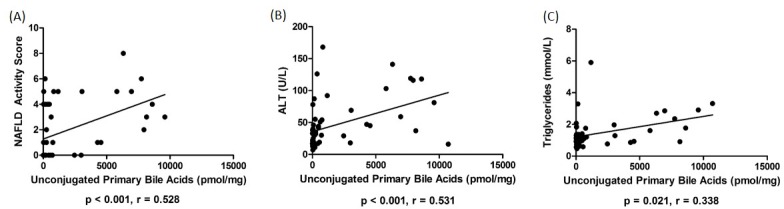
Correlations with fecal unconjugated primary bile acids. (A) Correlation of fecal unconjugated primary bile acids and NAS (p < 0.001, r = 0.528). (B) Correlation of fecal unconjugated primary bile acids and plasma ALT (p < 0.001, r = 0.531). (C) Correlation of fecal unconjugated primary bile acids and serum triglycerides (p = 0.021, r = 0.338).

Total fecal BA levels were higher in patients with NASH compared to HC (*p* = 0.006). The distribution of fecal BA is summarized in **Tables 2** and **3** and shown in **Fig 1**. Patients with NASH had a higher ratio of primary to secondary BA compared to HC. The ratio of total conjugated and unconjugated bile acids was not different between NAFLD and HC. Levels of unconjugated cholic acid (CA) were higher in both NASH and NAFL compared to HC, whereas chenodeoxycholic acid (CDCA) was higher in NASH vs. HC. The primary conjugated BAs were not different between the groups; however, patients with NASH had higher levels of glycine- and tauro-conjugated lithocholic acid (LCA) compared to patients with NAFL. Fecal levels of unconjugated primary bile acids positively correlated with steatosis, ballooning, fibrosis and NAFLD activity score (NAS) (*r =* 0.395, *p =* 0.011; *r =* 0.323, *p =* 0.042; *r* = 0.350, *p =* 0.029; *r =* 0.474, *p =* 0.002, respectively) (**Fig 2A**). Unconjugated primary bile acids also correlated with serum markers of liver injury AST, ALT, as well as triglyceride levels (*r =* 0.485, *p <* 0.001; *r* = 0.531, *p =* 0.000; *r =* 0.338, *p =* 0.021, respectively) (**Fig 2B and 2C**). Fecal levels of hyodeoxycholic acid (HDCA) inversely correlated with degree of steatosis and NAS (*r* = -0.419, *p* = 0.009; *r* = -0.551, *p* < 0.001).

**Table 2:** Unconjugated fecal bile acid composition and comparisons between the groups (n = 53).

Bile Acid (pmol/mg)	HC (n = 25)	NAFL (n = 12)	NASH (n = 16)	p-value
Primary BAs*	65 (7–10708)^a^	440 (25–8144)	997 (20–9592)^a^	0.002
CA*	28 (3–5392)^a,b^	255 (19–4196)^a^	391 (10–4916)^b^	0.001
CDCA*	45 (3–5316)^a^	196 (6–4023)	606 (9–4994)^a^	0.011
Secondary BAs	2063 (1490–5611)	2214 (5 3–3306)	1937 (40–4037)	0.753
DCA	1101 (614–3106)	1203 (12–1764)	1223 (16–2699)	0.959
LCA^ǂ^	952 (552–2505)	950 (9–1543)	908 (23–1338)	0.012
Primary to Secondary Ratio*	0.03 (0.0–2.5)^a^	0.1 (0.1–194.9)	0.4 (0.1–191.2)^a^	0.004
Total BAs*	2138 (1579–17192)^a^	3094 (2250–13062)	3833 (1374–14289)^a^	0.006

The values are expressed as medians (range).

^ǂ^ Significant comparisons between HC and NAFLD group (NAFL + NASH).

* Significant comparison between groups (*p* < 0.05).

For each comparison, identical letters indicate the groups between which the statistical difference was significant.

**Table 3:** Conjugated primary and secondary bile acid distribution in the study population (*n* = 53).

Bile Acid (pmol/mg)	HC(n = 25)	NAFL(n = 12)	NASH(n = 16)	*p*-value
Conjugated Primary	16 (2–391)	29 (3–175)	77 (4–4641)	0.137
Conjugated CA	8 (1–175)	19 (2–109)	49 (2–395)	0.098
Conjugated CDCA	8 (1–283)	13 (1–72)	19 (2–299)	0.197
Conjugated Secondary	18 (4–443)	21 (5–63)	66 (5–536)	0.249
Conjugated DCA	16 (2–422)	19 (2–48)	52 (4–426)	0.189
Conjugated LCA*	7 (1–150)	12 (1–18)^a^	13 (1–138)^a^	0.035

The values are expressed as medians (range).

For each comparison, identical letters indicate the groups between which the statistical difference was significant.

* Significant comparison between groups (*p* < 0.05).

### IM

In a subset of 29 participants (18 HC, 11 NASH) in which we were able to obtain IM data, patients with NASH had decreased counts of Bacteroidetes (HC = 8.48 vs. NASH = 7.77 log cell counts/g feces) and patients with NASH had decreased counts of *Clostridium leptum* (HC = 8.67 vs. NASH = 7.72 log cell counts/g feces) (*p* = 0.028 and *p* = 0.030, respectively) compared to HC, which remained significant after adjusting for BMI and weight-adjusted calorie intake. Bacteroidetes counts were inversely correlated with glycoLCA (*r* = -0.550, *p* = 0.002) and tauroLCA (*r* = -0.408, *p* = 0.048). *C*. *leptum* counts were positively correlated with fecal unconjugated LCA (*r* = 0.526, *p* = 0.003) and inversely with unconjugated CA (*r* = -0.669, *p* < 0.0001) and unconjugated CDCA (*r* = - 0.630, *p* < 0.0001). However, *C*. *leptum* counts were not significantly correlated with NAS score (*r* = -0.379, *p* = 0.109).

### C4 and FGF19

**Fig 3:**
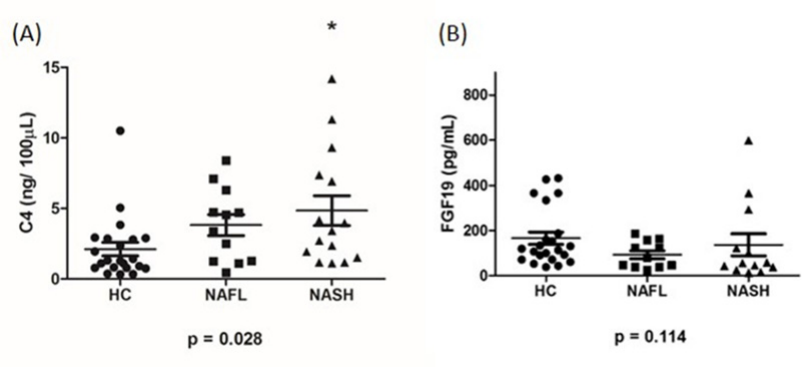
Serum C4 and FGF19 levels in HC, NAFL and NASH groups. (A) Serum C4 levels in HC, NAFL and NASH (*n* = 48) *p < 0.05 compared to HC. (B) Serum fibroblast growth factor 19 (FGF19) levels in HC, NAFL and NASH (*n* = 46).

Serum C4 and FGF19 data are summarized in **Tables 4 and 5**. Serum C4 levels were significantly elevated in NASH compared to HC, but was not different in NAFL (**Fig 3A**). C4 was also positively correlated with total fecal bile acid content (*r =* 0.337, *p* = 0.008). C4 was inversely correlated with FGF19 levels (*r* = -0.390, *p* = 0.028). There were no significant differences in serum FGF19 levels between patients with NAFL, NASH and HC (**Fig 3B**).

**Table 4:** Serum C4 in the study population (*n* = 48).

	HC (n = 22)	NAFL (n = 12)	NASH (n = 15)	*p*-value
C4 (ng/100μL) *	1.3 (0.3–10.5)^a^	4.0 (0.4–8.4)	3.4 (1.1–14.2)^a^	0.028

The values are expressed as medians (range).

* *p* < 0.05.

For each comparison, identical letters indicate the groups between which the statistical difference was significant.

**Table 5:** Serum FGF19 in the study population (*n* = 46).

	HC (n = 22)	NAFL (n = 11)	NASH (n = 13)	*p*-value
FGF19 (pg/mL)	116.6 (38.4–431.9)	100.5 (23.4–185.3)	56.9 (14.0–599.4)	0.114

The values are expressed as medians (range).

* *p* < 0.05.

## Discussion

Bile acids are key regulators of nutrient digestion and metabolism and their homeostasis is regulated by intestinal microbiota [30]. Dysbiosis has been reported in the setting of obesity, as well as in adults and children with NAFLD [9, 31–33], suggesting that IM may be contributing to the pathophysiology of this condition. In this study, we examined the fecal bile acid composition of patients with biopsy-confirmed NAFL and NASH, in conjunction with markers of bile acid synthesis and intestinal farnesoid X receptor (FXR) activation, and compared it against HC. We have demonstrated that patients with NAFLD, particularly those with NASH have higher total fecal bile acid levels, increased rates of bile acid synthesis in the liver and a predominance of primary bile acids in the stool that correlate with intestinal dysbiosis. This study provides the most comprehensive analysis of bile acid homeostasis in patients with NAFLD to date.

Elevated total bile acid levels have been previously observed in serum, plasma, urine and liver of patients with NAFLD, but fecal bile acids or rates of bile acid synthesis have not been previously studied in this setting [19–21]. In addition, the cause for the increase in total bile acid levels in all body compartments studied to date is not clear. In our study, total fecal bile acid levels were increased in patients with NASH, which is reflective of increased fecal bile acid losses. The increased rates of hepatic bile acid synthesis, indicated by higher levels of serum 7-alpha-hydroxy-4-cholesten-3-one (C4), a bile acid synthesis intermediate and a reliable marker of *de novo* bile acid synthesis [34], may represent the hepatic response to the increased fecal bile acid losses. It is not clear why patients with NASH would have decreased rates of bile acid reabsorption compared to healthy controls, especially considering that the ratio of conjugated to unconjugated bile acids was not different between the groups.

Evidence for increased bile acid synthesis in the setting of NASH has previously been reported by Lake *et al*. who found increased protein expression of bile acid synthesis enzymes in human NASH livers [35]. The increased bile acid synthesis in response to augmented fecal bile acid losses, does not explain however, why patients with NAFLD have previously been found to have increased bile acid levels in every body compartment studied [35]. A normal response to increased bile acid losses would be enhanced bile acid synthesis until the bile acid pool normalizes. The ongoing bile acid synthesis in the setting of increased bile acid levels may represent impairment in the regulation of bile acid synthesis, which may be FGF19 dependent or independent. In our study, there was no difference in FGF19 levels between patients with NAFLD and HC subjects. Previous studies of FGF19 levels in NAFLD have shown conflicting results. Some have shown similar FGF19 levels between patients with NAFLD and HC [36] and other have found reduced serum fasting FGF19 levels in NAFLD [37–39]. In addition, adults with NAFLD and insulin resistance have been previously found to have an impaired response to FGF19 [36]. In our study, the lack of difference in FGF19 levels may indicate a reason other than increased fecal bile acid losses for increased bile acid synthesis, such as increase of bile acid synthesis and in general, the dysregulation of cholesterol metabolism. Min et al. demonstrated that there was a decrease in CYP7A1 protein expression in NAFL and NASH compared to control, but there was a significant increase in mRNA expression of CYP7A1 in NAFL compared to lean control [40]. Cholesterol regulation remains an important area to be explored, particularly in the context of bile acid metabolism. Further studies are needed to describe the bile acid pool size in patients with NAFLD and to understand the role of FGF19 signaling in health and disease.

NASH is characterized by hepatic inflammation and often fibrosis, which may result from the hepatotoxic potential of bile acids [41]. In patients with NASH, fecal levels of cholic acid (CA), chenodeoxycholic acid (CDCA) and lithocholic acid (LCA) were increased. This finding is in agreement with previously published studies on the serum/plasma bile acid composition of patients with NAFLD [19, 21]. Interestingly, primary unconjugated fecal bile acids correlated with the degree of hepatic steatosis, the presence of ballooning, and the severity of fibrosis in patients with NASH. In rats, CDCA has also been shown to correlate with severity of hepatic steatosis in a high fat-cholesterol NAFLD model [42]. These findings may represent the hepatotoxic impact of hydrophobic bile acids, which include stellate cell activation and initiation of cell necrosis pathways [43–45]. Apart from direct hepatotoxic potential, bile acids can also contribute to the development of NAFLD through their effects on intestinal permeability. Primary bile acids increase intestinal permeability through epithelial growth factor receptor auto-phosphorylation, occludin dephosphorylation and re-arrangement of tight junctions [46]. Increased intestinal permeability has been linked to metabolic endotoxemia, insulin resistance and inflammatory cytokine release, and is a common finding in patients with NAFLD [47, 48]. The differences in fecal primary bile acid levels between patients with NAFL and NASH is an intriguing finding that should be investigated further, as it may help explain why some patients have NAFL while others develop NASH.

The altered bile acid profiles seen in patients with NAFLD may be the result of intestinal dysbiosis. In our study, unconjugated CA and CDCA were increased in NASH patients. Concurrently, patients with NASH had lower relative abundances of Bacteroidetes and *Clostridium leptum*, independent of BMI and weight adjusted caloric intake. *C*. *leptum* are among the few species in the colon that are capable of modifying bile acids by performing 7α-dehydroxylation and deconjugation, thereby converting primary bile acids to secondary ones [16, 49]. Our results show that the decrease in relative abundance of *C*. *leptum* is inversely associated with CA and CDCA, which may be illustrative *C*. *leptum*’s ability to convert primary to secondary bile acids [50].

Limitations of this study include its relatively small sample size. The small sample size of the NAFL group may not have been large enough to achieve statistical power. However, the sample size of the current study exceeds that of previous studies on bile acids and NAFLD and parallels those of other studies in metabolic diseases [38, 51]. With respect to the use of self-reported dietary data, this has been found to be limited reliability and in our study may reflect underreporting of food intake, particularly in patients with NAFLD. Alternatively, this may be reflective of increased energy harvesting capacity of the microbiome in patients with obesity [52]. In our study, healthy controls were not age-matched with patients with NAFLD due to the tendency for healthy controls to be younger as they were recruited from a living organ donation program. We do not believe the lack of age-matching to significantly affect our results as the healthy human adult microbiome is relatively stable during adulthood [53, 54]. While this study used quantitative polymerase chain reaction to identify specific bacterial strains of interest, future studies should aim to utilize more powerful next-generation 16S RNA sequencing techniques, such as Illumina [55].

In summary, patients with NASH have altered fecal bile acid profiles, characterized by increased bile acid synthesis and high fecal levels of cholic acid and chenodeoxycholic acid. Primary bile acids levels correlate with markers of hepatic injury as well as IM composition changes, suggesting a possible role for bile acids in the progression of NAFL to NASH. Further understanding the role IM and bile acids play in the pathogenesis of NAFLD may provide insights for the diagnosis of NAFLD as well as novel therapeutic targets for treatment.

## Supporting Information

S1 Dataset (XLSX)

## Abbreviations

NAFLD: non-alcoholic fatty liver disease
NASH: non-alcoholic steatohepatitis
NAFL: non-alcoholic fatty liver
HC: healthy control
IM: intestinal microbiota
BA: bile acid
FXR: farnesoid X receptor
IR: insulin resistance
ALT: alanine amino transferase
AST: aspartate aminotransferase
ALP: alkaline phosphatase
C4: 7-alpha-hydroxy-4-cholesten-3-one
LC-MS-MS: Liquid chromatography–tandem mass spectrometry
FGF-19: fibroblast growth factor 19
BMI: body mass index
HOMA-IR: Homeostasis Model Assessment of Insulin Resistance
HbA1c: Hemoglobin A1c
CA: cholic acid
CDCA: chenodeoxycholic acid
LCA: lithocholic acid
NAS: NAFLD activity score
HDCA: hyodeoxycholic acid
